# 

**Published:** 1899-07

**Authors:** 


*Fifty-Fourth Year.*

VOL. LIV.

WHOLE NUMBER,

DCXXXII.

JULY, 1899.

New Series.

VOL. XXXVIII.

No. 12.

### BUFFALO MEDICAL JOURNAL

**Established 1845 by AUSTIN FLINT, M. D.**

**EDITORS:**

THOS. LOTHROP, M. D. WM. WARREN POTTER, M. D.

**ASSOCIATE EDITORS:**

WILLIAM C. KRAUSS, M. D.

JAMES WRIGHT PUTNAM, M. D.

JOHN A. MILLER, PH. D.

JOHN PARMENTER, M. D.

ERNEST WENDE, M. D.

ALV[illegible] A. HUBBELL, M. D.

Index S. G. O.

LIBRARY
SURGEON GENERAL'S OFFICE
JUL.-3--1899

SCRIPTA FERUNT ANNOS

EUROPEAN AGENCY, J. B. BAILLIERE & FILS, 19 Rue Hautefeuille, Paris.
*Subscription, if paid in advance, $2.00; otherwise, $2.50. Foreign subscription, $2.50.*
Copyright 1899, by WILLIAM WARREN POTTER, M. D.
Entered at the Post Office at Buffalo, N. Y., as second-class mail matter.

A. T. BROWN PRINTING HOUSE, BUFFALO, N. Y.

**Table of Contents on Page V.**

